# Beyond the Gut, Emerging Microbiome Areas of Research: A Focus on Early-Life Microbial Colonization

**DOI:** 10.3390/microorganisms11020239

**Published:** 2023-01-18

**Authors:** Ravichandra Vemuri, Manoja P. Herath

**Affiliations:** 1 Department of Pathology, Wake Forest University School of Medicine, Winston Salem, NC 27101, USA; 2 School of Health Sciences, University of Tasmania, Launceston, TAS 7248, Australia

**Keywords:** microbiome, amniotic fluid, placenta, genitals, neonates, metabolic health

## Abstract

Undoubtedly, the human body harbors trillions of microbes of different kinds performing various physiological activities, such as priming the immune system, influencing host metabolism, and improving health by providing important metabolites such as short-chain fatty acids. Although the gut is considered the “microbial organ” of our body as it hosts the most microbes, there are microbes present in various other important anatomical locations differing in numbers and type. Research has shown the presence of microbes in utero, sparking a debate on the “sterile womb” concept, and there is much scope for more work in this area. It is important to understand the early-life microbiome colonization, which has a role in the developmental origins of health and disease in later life. Moreover, seminal studies have indicated the presence of microbes beyond the gut, for example, in the adipose tissue and the liver. However, it is still unclear what is the exact source of these microbes and their exact roles in health and disease. In this review, we appraise and discuss emerging microbiome areas of research and their roles in metabolic health. Further, we review the importance of the genital microbiome in early-life microbial interactions.

## 1. Introduction

While there are still inconsistencies about who was the first to coin the terms microbiota and microbiome, it is accepted that “microbiota” is a collection of microorganisms in a defined environment. The most accepted definition of the term “microbiome” is the collection of all the microbial genes or genomes of the microbiota [1]. Others in the field extend the microbiome definition to the entire habitat, including bacteria, viruses, fungi, archaea, and others [2]. Moreover, a few utilize the term “metagenome”: a collection of genes and genomes of the microbiota, which comes from molecular methods such as “metagenomics” [1,2]. Metagenomics methods, such as 16S rRNA or Whole Genome Sequencing (WGS), are the next-generation sequencing methods to get microbial information from a given biological sample [3]. Moreover, this sequencing allowed the sampling of the principal multi-kingdom species present within a microbiome. Each sequence is assigned to individual microbial taxon such as bacteria, viruses, fungi, and archaea from phylum to species taxonomic levels. The novel terms with respect to an individual microbial taxon that are emerging in microbiome research include “bacteriome” (bacterial communities), “virome” (viral communities) including phageome (phage communities), “archaeome” (archaeal communities), and “mycobiome” (fungal communities) [3]. All these multi-kingdom microbial communities interact with each other and play an important role throughout the human lifecycle, even before birth. The long-standing sterile womb hypothesis is challenged by emerging research, altering our previous understanding of microbiome establishment after birth [4,5,6]. These studies have identified microbes and their products in amniotic fluid, placenta, and meconium, demonstrating the in utero microbial establishment, which continues during the first three years of life. However, the sources of these in-utero microbes and the routes by which they access the fetal gut remain largely unknown. A few studies have indicated that these microbes are maternally sourced mainly due to bacteria found in cord blood, however, the root can go back to genital microbiomes [7,8,9]. Perturbations in these different biomes during prenatal to neonatal periods can have short- and long-term consequences in an infant’s life, such as preterm birth, autoimmune disorders, chronic lung disease, inflammatory bowel diseases, eczema, asthma, obesity, and central nervous system disorders. 

**Figure 1:**
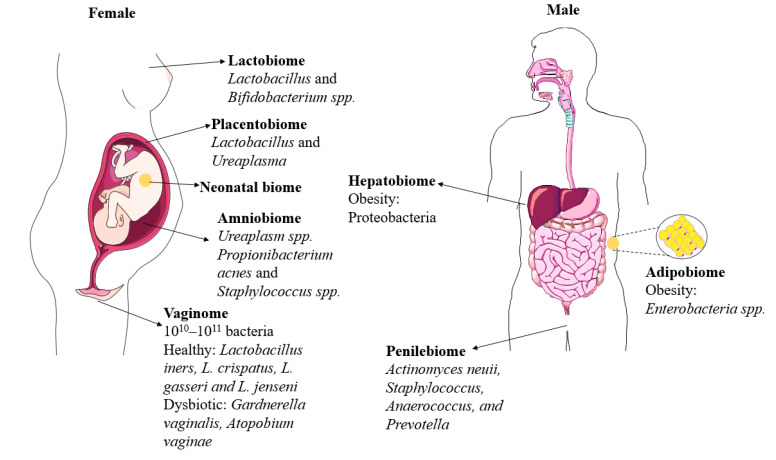
Overview of emerging biomes. Beyond the gut, emerging microbiomes in human body sites (in both males and females) influence overall health and well-being. This figure provides information about the bacterial microbiome.

The purpose of this review is to: (i) review novel areas of microbiome research beyond the gut, along with a few new “biotas” and “biomes” for the very first time (Figure 1), and (ii) provide a novel perspective on developmental origins of the microbiome and concisely discuss the importance of research beyond the gut microbiome. 

## 2. Genital Microbiomes

### 2.1. Vaginobiome

The terms vaginobiota and vaginobiome (or vaginome), for very first-time use, describe the collection of microbial communities and their genome of the vaginobiota in the female reproductive tract. Vaginobiome is a dynamic micro-ecosystem, which is constantly influenced by the menstrual cycle and immune system, and this bi-directional crosstalk is known as microgenderome [10,11,12]. In healthy females, *Lactobacillus* spp. is dominant in the vaginobiome [13] (Table 1). A variety of microbes are transferred from male to female partners during copulation and dysbiosis in the vaginobiome, which can be linked to intrauterine microbial sources [14]. Understanding the genital microbiomes can lead to understanding sexually transmitted infections or related health outcomes and research into prevention.

### 2.2. Penilebiome

The terms penilebiota and penilebiome, for very first-time use, describe the collection of microbial communities and their genome of the penilebiota in the male reproductive tract. *Actinomyces neuii*, *Staphylococcus, Anaerococcus*, and *Prevotella* are the genera that are more abundant over time [14,16].

## 3. Microbiomes Associated with the Early Life Development

### 3.1. Amniobiome

The terms amniobiota and amniobiome, for very first-time use, describe the collection of microbial communities and their genomes of amniobiota in amniotic fluid. A multitude of studies suggests the presence of microbes in utero employing advanced next-generation sequencing technologies, which is a paradigm shift in the idea of a sterile intrauterine environment [5,7,8]. Specifically, *Ureaplasma* spp. was detected in the amniotic samples at 16–20 weeks gestation and post-third trimester [17]. Propionibacterium acnes and Staphylococcus spp. are the few other species reported in amniotic fluid. In addition, bacterial communities were identified in the meconium and the placenta concluding that the microbial colonization of the fetal gut begins in utero and continues during the first two years of life [5,18]. Indeed, alteration of the placental and amniotic fluid bacteriome has been associated with preterm birth [26].

### 3.2. Placentalbiome

The collection of microbes in the placenta can be termed placentalbiota, and their genomes together are termed placentalbiome. The placenta is an exchange site of nutrients and blood between the fetal and maternal systems and performs vital metabolic functions supporting fetal development and maintaining maternal-fetal tolerance [27]. Interestingly, *Lactobacillus* and *Ureaplasma* spp. were most commonly found in the placenta [19,28], where *Lactobacillus* spp. was associated with the healthy human gut, breast milk, and vaginome.

### 3.3. Meconiobiome

The collection of microbes in meconium can be termed meconiobiota, and their genomes together are termed meconiobiome or meconiome. Meconium is the first postnatal bowel movement of infants. The post-birth meconium microbiome is thought to represent the in-utero microbial environment [18]. Regardless of whether meconium is colonized before, during, or after birth, the composition and structure of early-life gut communities may influence health later in life. Meconium mostly shared dominated by *Lactobacillus*, *Bifidobacterium, Staphylococcus*, and *Enterococcus* spp. [15].

### 3.4. Lactobiome

Besides the mode of delivery and environment, early nutrition through breastfeeding is a key factor directing the neonatal microbiota composition. The collection of microbes in breast milk can be termed lactobiota, and their genomes together are termed lactobiome. Breastmilk is considered a seed microbiome in an infant’s gut, and the components of milk nurture the microbiome with beneficial bacteria. Lactobiome is mostly dominated by beneficial bacteria such as *Lactobacillus* and *Bifidobacterium* spp. [20,21]. Beneficial gut microbes play a role in lowering the risk of later-life chronic diseases like asthma, obesity, allergies, dermatitis, inflammatory bowel disease, and neurodevelopmental disorders [29].

## 4. Microbiomes Associated with Later-Life Metabolic Health: Emerging Research Areas

For a long period, metabolic tissues such as adipose and liver tissues were considered sterile sites and, as such, were not examined for the presence of the microbiome. However, emerging studies have clearly demonstrated the presence of microbes in metabolic tissues.

### 4.1. Adipobiome

Recently, a few studies have identified bacterial signatures in metabolic tissues, such as adipose tissue and the liver. The collection of microbes in the adipose tissue can be termed adipobiota, and their genomes together are termed adipobiome. Human studies demonstrated an association of certain Proteobacteria phylum members, such as *Enterobacteria* spp., to the initiation of subclinical inflammation, influencing metabolic pathways leading to obesity [22,23]. Although the physiological role and source of these bacteria are still under debate, microbial translocation is identified as the viable route. In addition, early-life exposure to microbes in the womb might be an interesting hypothesis for the microbial source in the metabolic tissues [22,30].

### 4.2. Hepatobiome

Similar to the adipobiome, the collection of microbiota in the liver can be termed hepatobiota, and their respective genome is termed heptabiome. Suppli et al. and a few other researchers examined liver biopsies from lean and obese individuals. They found that the composition of bacterial DNA in the livers of the obese group differed from the lean group [24,25,31]. In addition, heptabiome followed similar patterns of higher Proteobacteria phylum in the obese group, suggesting a liver-adipose axis in the development of the metabolic syndrome. Leinwand et al. also found the presence of heptabiome in mice and humans, where higher abundances of Proteobacteria induced inflammation [25]. These findings provided a rationale for microbiome-based therapies in treating liver disease.

## 5. Discussion

**Figure 2:**
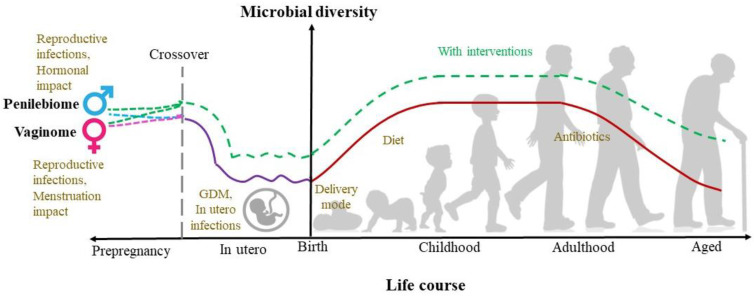
The potential relationship between microbial diversity and age in humans. The red line represents the well-established phases of the human microbiome [34,35,36,37,38,39]: the establishment or developmental phase during birth, the transitional phase during infancy, the stability phase during adulthood, and the decline phase in aged and centenarians. The purple line represents in utero microbial colonization [5,7,15,40]. The green line represents how early interventions (e.g., probiotic supplementations) could reduce the decline in microbial diversity with age [41,42].

The microbiome comprises a diverse collection of bacteria, viruses, fungi, archaea, and others. A normal, healthy microbiota allows the maintenance of immune homeostasis, but dysbiosis (the imbalance of microbial species with reductions in beneficial microbiota and increases in harmful microbiota) can drive inflammation and susceptibility to inflammation and various diseases. Microbiome development can be categorized into four phases: the establishment or developmental phase, transitional phase, stability phase, and decline phase [32]. A growing body of research has detected microbes in the placenta and amniotic fluid in normal and uncomplicated pregnancies, suggesting a step back in the establishment phase of the womb [33]. In Figure 2, we propose the potential relationship between microbial diversity and age in humans. The well-researched model categorized gut microbial changes into four phases: the establishment or developmental phase during birth, the transitional phase during early infancy up to 2–3 years, the stability phase during adulthood, and the decline phase in aged and centenarians. The emergence of the in-utero microbial colonization hypothesis provides some evidence of in-utero microbial exposure and the road to initial colonization. Gestational diabetes mellitus, in-utero infections, and the role of reproductive health from the pre-pregnancy stage can influence microbiome composition and diversity, leading to various health conditions from infancy to old age. Early life interventions remain crucial to change unhealthy microbiome composition trajectory (green dotted line), and recent research has shown that microbes impact the early life microbiome in different anatomical locations of the human body. Here, the author proposed a predicted model of microbial exposure to understand the involvement of the penilebiome and vaginome from the point of copulation where microbial crossover occurs and, therefore, possibly the road to initial colonization. Reproductive biomes are influenced by various factors; in males, they are sexually transmitted infections and testosterone levels. Here, the vaginome could play a vital role and is also affected by infections (bacterial vaginosis and viral infections) and the menstrual cycle (estrogen and progesterone). Microbiome-altering intervention during pre-pregnancy (in both males and females) studies using specific probiotics and care will be crucial.

On the contrary, many studies oppose the in-utero microbial colonization hypothesis [43,44]. The most important reasons are the accuracy of demarcating the microbial profiles using 16S rRNA sequencing technology and diversity at low biomass from these studies was hindered by our ability to distinguish the authentic signals beyond the level of background contamination, as the profiles were comparable to positive and negative controls [22,29,45]. Further, a conclusion that no microorganisms were present in the mid-trimester amniotic fluid of healthy pregnancies was reached using NGS techniques [43,46]. However, this is not in the case of low gestational age delivery and preterm births, which have shown bacterial infections in amniotic fluid [47,48].

To date, the opportunistic bacterial colonization of the amniotic fluid has been well documented and thoroughly studied in the context of spontaneous preterm delivery and fetal infection. Intrauterine infections trigger spontaneous preterm labor, accounting for two-thirds of all preterm births. It is one of the leading factors in the development of perinatal autoimmune and childhood neurological problems [29]. In addition to preterm births, bacteria were also found in healthy meconium and umbilical cord blood [33]. Both meconiobiome and cord blood shared microbe species with the microbiota of amniotic fluid, maternal feces, and placenta [7,11,15,18,49]. These findings indicate that the in-utero environment is not necessarily sterile, even in normal pregnancies, and show the association between preterm deliveries and infection. However, it is unclear when microbes first can access and occupy the amniotic space, and the sources of bacteria and routes by which they access fetal developmental times remain largely unknown.

Concerning the role of the menstrual cycle and in-utero bacterial colonization, research provides additional evidence of diverse bacteriomes in the female reproductive tract or the vaginome [50]. It has been suggested that certain obstetrical and neonatal complications are linked to maternal vaginome dysbiosis originating from asymptomatic infections, such as chronic endometritis, probably arising prior to conception [51]. As mentioned in the above sections, a few studies have identified differences in the vaginal microbiomes of pregnant and non-pregnant women [52]. These studies have shown that the pregnant vaginome is less rich and less diverse as compared to the non-pregnant vaginome, with a high proportion of *Lactobacillus* spp. [53,54]. Important factors such as behavioral changes, physio-chemical changes in the mucosa, hormonal changes leading to immune modulation, and changes in the genital tract may define the modulation in structure and function of the vaginome, making it unique from non-pregnant females. There is ample evidence demonstrating the associations between vaginome dysbiosis and preterm birth. Vaginome dysbiosis is caused mainly by bacterial vaginosis, where Firmicutes and Actinobacteria phyla were identified as causative bacteria [13,14,16,54]. Bacterial vaginosis in early pregnancy is linked to preterm or delivery of a low birthweight infant [55]. This is because bacterial vaginosis-causing microbes (*Gardenella vaginalis*, *Sneathia sanguinegens*, *Atopobium vaginae*, and *Mobiluncus curtsii*) might cause infection during gestation because they can move into the uterus before gestation. Similar to gut dysbiosis, an association is made between vaginome dysbiosis (decreased abundance of lactobacilli and increased *Gardenella* spp.) and preterm birth and infertility [11,56]. Particularly, *Lactobacillus* spp. is a lactic acid producer, maintaining lower pH (~3.5) in the vaginal environment and producing bacteriocins protecting from opportunistic pathogen colonization [53]. The identified research gaps include the role of host and vaginal mucosa in health status-based selective colonization of the microbiome. Deciphering the relationship between the vaginome, host, and the immune system can even provide therapeutic intervention strategies, for instance, pro- or antibiotics that might benefit maternal health. Further, daily changes in the vaginome have been identified, and of these changes, maximum changes occurred during menstruation, followed by copulation [57].

Research on the male genital microbiome is emerging [14,16]. The penile body site is a reservoir of microbes and can carry bacteria that cause vaginal infections. Zozaya et al. demonstrated that copulation is a major source of the interchange of the microbiome and is a risk factor for bacterial vaginosis and dysbiosis [58]. This interchange of biome may have an impact on preterm and gestational age and infant birth weight. More studies are required in this area of research to better understand the impact of the microbe interchange during copulation on the amniotic and placental microbiome.

As discussed earlier, in the developmental and stability phase of the human lifecycle, skin (dermabiome), breast milk (lactobiome), and oral microbiome are the significant biomes influencing the structure and development of the whole body microbiome [32]. Immediately after birth, bacterial communities on infant skin are undifferentiated and represent a combination of vaginal (vaginal delivery), skin, and environmental biomes (C-section) depending on the mode of delivery [59]. *Propionibacteria acnes* is found to be the major microorganism and is stable over time up to the first 3 years of life. Most of the adult skin biomes show relatively more sebaceous, moist, or dry conditions [60]. Timely and proper skin bacterial establishment during early life might have a vital protective role against infections in childhood and later life. Other species, such as *Staphylococcus epidermidis* and *Staphylococcus aureus* were well studied to understand microbe-skin immune interactions. These dermabiomes, similar to the gut microbiome, contribute to the establishment of skin-immune homeostasis. Healthy dermabiome can produce molecules that can inhibit pathogen colonization on the skin. Overall, the skin, similar to the gut and other biomes, harbors a diverse community of microorganisms that each have distinct adaptations to survive on the skin.

Breast milk is considered one of the first sources of neonatal gut microbial colonization post-delivery [61]. The gut of infants who were breastfed predominantly comprised streptococci and bifidobacteria, and in formula-fed infants, the gut was dominated by Enterobacteria and Bacteroides [61,62]. These differences may have metabolic and immune implications affecting the overall health of the infant. Similarly, it has been suggested that microbes that reside in the oral cavity (oral microbiome) change with age and are influenced by feeding type [63]. The oral microbiome is not similar to the individual’s gut microbiome, and the dominant bacteria are Firmicutes, Proteobacteria, Bacteroidetes, Actinobacteria, Fusobacteria, Tenericutes, and TM7 [64]. Further, these above-mentioned heterogeneous microorganisms harbor in the oral cavity at different anatomical sites, which is significant for homeostasis with the host immune system. The clinical relevance of the oral microbiome findings and their health implications need to be further explored. Currently, lactobiome research (which has implications for oral microbiome) is focused on finding answers for: (a) the role lactobiome bacterial communities play on infant health and development, including their influence on the establishment of the neonatal microbiome [61]; (b) the roles that they may play on maternal health, including breast health; (c) their origin, in order to accept or reject the existence of an endogenous oral-gut-mammary route allowing the selective translocation of some bacteria from the maternal digestive tract to the mammary gland [65]; and (d) its impact on metabolic health in infants and later life.

Being overweight and obese is a significant risk factor for several life-threatening metabolic diseases such as cardiovascular diseases and type 2 diabetes mellitus (T2D) [66]. The life course approach describes that the risk of metabolic diseases increases throughout life, starting from the intrauterine period. This increase in the risk of metabolic diseases has been explained as a consequence of the decline in plasticity, where one genotype forms different physiological or morphological states in response to influences of environmental conditions [67]. In humans, plasticity is at its maximum during the first 1000 days of life, the period from conception to two years of age. During this period, most of the biological development is completed [68]. Once the offspring adapts its growth trajectory in the time of fetal life and early infancy, it is relatively irreversible [69]. Along these lines, a stimulus that results in excess accumulation of adipose tissue in-utero or early infancy may predispose individuals to obesity in later childhood and adulthood [70]. Since the mother provides the intrauterine environment for the developing fetus, it is expected that the nutritional, endocrine, behavioral, and environmental factors of the mother during pregnancy are reflected in the metabolic health of the infant at birth [71]. Studies have found associations of gestational diabetes mellitus with concordant alterations in maternal and neonatal metabolic health and microbiotas [72,73]. During the postnatal period, infant feeding practices play the most important role. Among the numerous health benefits of breastfeeding is the reduced risk of future obesity. This protective effect of breastfeeding may partly be moderated due to its impact on infant microbiota colonization and development [74].

A wealth of research demonstrated that the changes in the gut microbiota of an individual are associated with obesity and T2D conditions; recent research has shown microbial colonization in the liver and adipose tissues [22,23]. Energy metabolism is key to obesity and governed by metabolic organs such as the liver, which in turn metabolically connect with adipose tissue. These studies on hepatobiome and adipobiome have shown microbial signatures different from the gut and their relevance to metabolic or other potential diseases. Similar to the studies currently demonstrating that the womb is not sterile anymore, a study by Branton et al. provided some evidence of microbes in the brain (neurobiome) of HIV/AIDS patients [75]. Utilizing the NGS technology, they found the presence of specific bacteria and phages in the brain. Although this concept has emerged, the contention that the human brain is sterile has not been well tested. Further research on these “game-changing” findings is needed to understand the origin and source of these signals (ruling out contamination) would be of interest. 

With the gut microbiome research, we know the microbiome composition is altered by factors such as antibiotic usage, travel, diet, environment, and age [32]. The colonization of these microbiomes on the different parts of the body is determined not only by the above-mentioned factors but is also modified by factors that impose selective pressure on the body microbiotas, such as host genetics and vertical transmission from mother to infant. A new hypothesis can be generated on the role of the genital and in-utero biome in the metabolic tissue microbiota. Further, all the research in this area has focused exclusively on evaluating the existence of bacterial communities in the fetal and placental tissues. The existence of eukaryotic microbial communities and viruses (phages) in these tissues was not considered [3,32]. Although bacteriophages were not identified as human disease-causative microbes, they play a major role in the causation of many infections by influencing the growth of the bacteriome [76]. A few studies have managed to sequence viromes along with bacteriomes; however, their works were limited in not opting for whole genome sequencing (WGS) to generate a full-coverage genome sequence [3]. Therefore, well-controlled studies are needed with proper essential controls and multi-omics analyses to substantiate the microbial presence in the placenta. Particularly on the microbiome front, studies that employ the WGS technology to get the multi-kingdom strain-level resolution profiles at low biomass are required.

## 6. Conclusions and Future Directions

The human microbiome is a diverse ecosystem, and the notion that the gut is the microbial organ is evolving. Research into the human microbiome has been increasing exponentially in recent years. In fact, such is its importance that it is considered as an organ itself: the so-called “forgotten organ” [77]. Emerging microbiome research areas beyond the gut that are addressed in our review include the placenta, amniotic fluid, cord blood, breast milk, and metabolic tissues. These locations are compartmentalized in a unique fashion where the microbial composition is less shared with the gut microbes. That is, specific microbes in each anatomical location perform different physiological functions. The origins of many diseases are hugely influenced by the health of the mother, and commensals and opportunistic microbes clearly play a major role. 

Furthermore, we consider the following points as crucial for identifying microbial establishment in the human lifecycle:

(i) Microbial progression phases during a human lifecycle, as mentioned in the earlier sections, may not be established post-birth. Emerging research challenges the sterile womb hypothesis with evidence of the microbial presence in various sites in the womb.
(ii) The role of contamination is paramount in the identification of the true representation of the “microbiome” due to their low biomass. Low biomass data is impacted by analyzing a relatively small sample size. The small sample size or underpowered microbiome research can reduce the amplification of low biomass samples, in turn lowering the detection sensitivity and resolution. To gain more insight into bacteriomes, a more robust and optimized 16S gene sequencing pipeline with longer reads will be beneficial to catalog the bacterial DNA profiles of different tissues and provide a database to analyze host/bacterial interactions in relation to homeostasis and disease.
(iii) The reasons for low biomass in the observed bacteriome in amniotic fluid passed via the maternal, as seen in cord blood, but may not be able to survive the womb due to host defenses but be transient in nature.
(iv) Another interesting reason could be the presence of microbes beyond bacteriomes, such as viromes. Virome majorly includes phages, which may play a role in maintaining low biomass and the lack of proper colonization, such as the gut. NGS technologies such as WGS should be utilized, which is a more robust and high-resolution platform.
(v) A more important aspect to consider is the role of female health, not only during pregnancy and post-pregnancy but also during pre-pregnancy and copulation. Identification of vaginome and penilebiome has revolutionized microbiome research and can provide information on possible in-utero infections, preterm labor, and microbial sources. These genital biomes should be considered important in understanding the microbiome establishment, which will provide scope to modulate these biomes, in turn, neonatal health.
(vi) During the transition and stable phases, the lactobiome plays a key role in growth and metabolism. More research into the lactobiome-hepatobiome-adipobiome axis will be beneficial in understanding the future risk of metabolic diseases.
(vii) As the “omics” technologies are getting updated as we speak, more research utilizing advanced sequencing methods (proteomics, transcriptomics, metabolomics, RNA-seq, and Immuno-seq) is necessary to understand the in-utero colonization, which perhaps will enable us to prevent the origins of many diseases.

Application of these clarifications and recommendations may enable researchers to design microbiome studies in a holistic way, which will help to develop microbial models and predictions, which in turn will accelerate our ability to design applications in all areas of microbiome research.

## Figures and Tables

**Table 1:** Overview of microbiome clinical studies other than gut discussed in the paper.

Biome	Study Type	Dominant Bacteria	Method Used	Reference
1. Vaginome	Human (pregnant and non-pregnant)	Pregnant: *Lactobacillus* vagitypes (*L. crispatus*, *L. iners*, *L. gasseri* and *L. jensenii*) Non-pregnant: *Lactobacillus*	V1–V3 16S rRNA	[11]
	Vaginal microbial profiles in European (E)	Pre-pregnancy: *L. crispatus* Pregnancy: *L. jensenii*, *L. crispatus* PP: BV- *associated taxa Prevotella* spp., *Clostridium* spp., *Atopobium* spp. and *Megasphaera* spp.	V1-V2 16S rRNA	[12]
	Vaginal microbial profiles in African American (AA) versus European (E) ancestry women	BV: *Gardnerella vaginalis* (AA) AA: *L. iners* E: *L. crispatus*, *L. iners*, *G. vaginalis* AA/E differences: *Mycoplasma*, *Gardnerella*, *Prevotella* and *Sneathia*	V1–V3 16S rRNA	[13]
	Vaginome during pregnancy, preterm and PP	Pregnancy: *L. crispatus*, *L. gasseri*, *L. iners*, *L. jensenii* Preterm: *Gardnerella* and *Ureaplasma* PP: *Peptoniphilus*, *Prevotella*, and *Anaerococcus*	V3–V5 16S rRNA	[15]
2. Penilebiome	Penile (both meatal and glans/coronal sulcus/circumcised) and vaginal microbial profiles related to BV	Penile: *Corynebacterium* (circumcised), *Streptococcus*, *Anaerococcus*, *Finegoldia*. BV: *Parvimonas*, *L. iners*, *L. crispatus*, *Fastidiosipila,* and *Prevotella*	V3–V4 16S rRNA	[14]
	Vaginal and penile microbiomes related to herpes simplex virus type 2 (HSV-2)	BV: *G. vaginalis* and *L. iners* Penile: *Ureaplasma* and *Aerococcus* (HSV-2)	V3–V4 16S rRNA	[16]
3. Amniobiome	Preterm in 2nd trimester, asymptomatic	*Ureaplasma* and/or *Mycoplasma* spp.	16S rRNA	[17]
	In utero to first 4 days of birth	*Enterobacter*, *Escherichia/Shigella* and *Propionibacterium*	V1-V3 16S rRNA	[5,18]
4. Placentalbiome	In utero to first 4 days of birth	*Propionibacterium, Enterobacter and Escherichia/Shigella*	V1-V3 16S rRNA	[5]
	Meconium in twins	*Salinibacter* and Enterobacteriaceae_unclassified	V3-V4 16S rRNA	[19]
5. Meconiobiome	In utero to first 4 days of birth	*Propionibacterium, Escherichia/Shigella*, and *Lactobacillus*	V1-V3 16S rRNA	[5]
	Meconium in twins	Enterobacteriaceae_unclassified	V3-V4 16S rRNA	[19]
	Temporal and spatial variation in early-life microbiome	*Lactobacillus*, *Bifidobacterium, Staphylococcus*, and *Enterococcus* spp.	V3–V5 16S	[15]
6. Lactobiome	Milk microbiome	*Staphylococcus* and *Streptococcus*	V1-V3 16S rRNA	[20]
	Milk microbiome	*Streptococcus*, *Staphylococcus*, *Serratia* and *Corynebacteria*	V1-V2 16S rRNA	[21]
7. Adipobiome	Adipose tissue microbiome	Proteobacteria and Firmicutes	V4-V5 16S rRNA	[22]
	Adipose tissue microbiome related to type 2 diabetes (T2D) and obesity humans	*Pseudomonas, Faecalibacterium, Bacteroides* and *Enterobacter*	V3-V4 16S rRNA	[23]
8. Hepatobiome	Liver microbiome in obese and non-obese humans	Obese: Proteobacteria, *Massilia* spp.	V3-V4 16S rRNA	[24]
	Liver tissue microbiome related to diabetes and obesity humans	Obese: *Pseudomonas, Arthrobacter* and *Ruminococcus*	V3-V4 16S rRNA	[23]
	Liver microbiome in Humans and Mice	Mice: *Pseudomonas, Delftia* and *Coprococcus* Humans: Proteobacteria	16S rRNA	[25]

PP, postpartum; BV, bacterial vaginosis.

## Data Availability

Not applicable.
